# Corticotroph Tumour Type Influences Clinical Behaviour in Patients With Nonfunctioning Pituitary Neuroendocrine Tumours

**DOI:** 10.1002/edm2.70143

**Published:** 2025-12-05

**Authors:** Nasrin Al‐Shamkhi, Britt Edén Engström, Olafur Gudjonsson, Johan Wikström, Olivera Casar‐Borota, Eva Rask

**Affiliations:** ^1^ School of Medical Sciences, Faculty of Medicine and Health Örebro University Örebro Sweden; ^2^ Endocrinology and Mineral Metabolism, Department of Medical Sciences Uppsala University Uppsala Sweden; ^3^ Neurosurgery, Department of Medical Sciences Uppsala University Uppsala Sweden; ^4^ Neuroradiology, Department of Surgical Sciences Uppsala University Uppsala Sweden; ^5^ Genetics and Pathology, Department of Immunology Uppsala University Uppsala Sweden; ^6^ Department of Clinical Pathology Uppsala University Hospital Uppsala Sweden

**Keywords:** hypopituitarism, pituitary neuroendocrine tumour, transcription factors

## Abstract

**Introduction:**

This study aims to describe whether the clinical behaviour of nonfunctioning pituitary neuroendocrine tumours/nonfunctioning pituitary adenomas (NF‐PitNETs/NFPAs) is affected by pituitary cell lineage differentiation, focusing on patients with silent corticotroph tumours (SCTs) and silent gonadotroph tumours (SGTs).

**Methods:**

Patients (*N* = 101) who underwent primary surgery for NF‐PitNETs/NFPAs from August 2014 to March 2020 at Uppsala University Hospital were included. Data on sex, age, MRI, pituitary function and immunohistochemical analysis of anterior pituitary hormone and transcription factor expression, were explored.

**Results:**

Seventy‐three patients had SGTs, and 18 had SCTs. Binary logistic regression revealed that having SCT versus SGT (OR 6.41 (CI: 1.20–34.42), *p* = 0.03), being older at the time of surgery (OR 1.07 (CI: 1.02–1.12), *p* = 0.01), and having a larger preoperative tumour volume (OR 1.17 (CI: 1.04–1.32), *p* = 0.01) were associated with an increased likelihood of postoperative pituitary failure. Patients with preoperative pituitary failure were older at the time of surgery (*p* = 0.01) and more often had preoperative elevation of prolactin levels (*p* = 0.01) than patients without preoperative pituitary failure. SCT patients were younger at the time of surgery than SGT patients (*p* = 0.003), but no significant difference in preoperative tumour volume was detected.

**Conclusion:**

The results indicate that cell lineage differentiation in NF‐PitNETs/NFPAs influences clinical behaviour. Patients with SCTs were younger at the time of surgery, and harbouring a SCT was associated with an increased likelihood of having postoperative pituitary failure. These findings emphasise the importance of routine immunohistochemical analyses of anterior pituitary hormone and transcription factor expression to identify silent corticotroph tumours.

## Graphical abstract

Harbouring a silent corticotroph tumour (SCT) compared to a silent gonadotroph tumour, being older at surgery and having a larger preoperative tumour were found to increase the likelihood of having postoperative pituitary failure in logistic regression analysis (*n* = 77). SCT patients were younger at surgery, albeit not having larger tumours preoperatively.

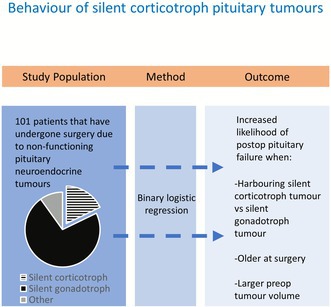

## 1 Introduction

According to the 2022 World Health Organization (WHO) classification of endocrine tumours, adenohypophysial tumours, including nonfunctioning tumours, are classified based on pituitary cell lineages, which are defined by immunohistochemical (IHC) analysis of anterior pituitary hormone and pituitary‐specific transcription factor (TF) expression [1]. TFs regulate differentiation into separate cell lineages in the anterior pituitary gland [2]. The T‐Box family member TBX19 (TPIT) is a TF that regulates differentiation into the corticotroph cell lineage, steroidogenic factor‐1 (SF1) is expressed in cells from the gonadotroph cell lineage, and pituitary‐specific positive transcription factor 1 (PIT1) is a TF that regulates differentiation into the somatotroph, lactotroph, and thyrotroph cell lineages [1]. Adding TF analysis to the IHC work‐up has decreased the number of nonfunctioning, hormone‐negative, and transcription factor‐negative “null‐cell” tumours to less than 2% [3, 4]. There has also been a proposal for a change in terminology from pituitary adenoma to pituitary neuroendocrine tumours [5], which has sparked a debate [6], leading to the parallel use of the terms pituitary neuroendocrine tumours/pituitary adenomas [1]. In the following report, focusing on cell lineage, the term nonfunctioning pituitary neuroendocrine tumour (NF‐PitNET) is chosen.

NF‐PitNETs are common pituitary lesions [7, 8], and a prevalence of up to 41.32/100000 has been reported [9]. The most common subtype is silent gonadotroph tumours (SGTs), which account for more than 70% of these lesions, followed by silent corticotroph tumours (SCTs) [2, 10]. According to the 2022 WHO classification, SCTs are among the potentially more aggressive subtypes of NF‐PitNETs, i.e., with a greater potential for recurrence and rapid or invasive growth [11]. Factors beyond the histological type that are assessed in the prediction of pituitary tumour behaviour include tumour size, invasiveness, levels of the proliferation marker Ki67, the mitotic count, and nuclear staining for p53 [12, 13].

NF‐PitNETs often cause pituitary failure; 60%–85% of patients with macroadenomas (≥ 1 cm in largest diameter) are deficient in at least one pituitary axis at diagnosis [14].

Data about the degree to which preoperative pituitary failure can be reversed after tumour surgery is conflicting, and new hormonal deficiencies can occur postoperatively [15, 16, 17]. The extent to which the pituitary cell lineage influences the clinical picture is not fully known [18]. Pooled data from a literature review that included studies, regardless of whether they comprised transcription factor analysis or not, revealed preoperative hypopituitarism in 38% of patients harbouring SCTs (range 11%–76%) [19]. One study displayed a greater degree of preoperative pituitary failure in patients with SCTs than in non‐SCT patients [20], whereas other studies have not shown such differences between SCT and other NF‐PitNETs or SGTs in particular [21, 22, 23]. New‐onset postoperative pituitary failure was more common in patients with SCTs than in patients with SGTs in some but not all studies [22, 24, 25].

In this study, we aimed to evaluate whether pituitary cell lineage differentiation in NF‐PitNETs, with a focus on patients with SCT and SGT, is linked to clinical behaviour at presentation and postoperatively, which consequently can affect the pre‐ and postoperative management of these patients.

## 2 Material and Methods

### 2.1 Patient Cohort and Clinical Data

All patients (*N* = 115) who had undergone primary surgery due to NF‐PitNETs from August 2014 to March 2020 at Uppsala University Hospital were invited to participate in the study. Thirteen patients declined to participate or did not respond to the invitation, and one patient was deceased; thus the cohort included 101 patients. Written informed consent and tumour tissue samples were obtained through the university‐initiated U‐CAN project (www.u‐can.uu.se) [26].

All the patients had histopathology reports confirming the PitNET diagnosis. All the surgeries were performed by one experienced neurosurgeon (OG). None of the patients had received radiotherapy prior to primary surgery. Patient data were included for the above‐mentioned time period or until the patient received a second treatment (10 patients).

Data on age at the time of surgery, sex, and pre‐ and postoperative pituitary function were retrieved from medical charts. Postoperative data were collected until January 2021 or the patient received a second treatment. Laboratory results regarding pre‐ and postoperative pituitary failure and hormone replacement therapy were re‐evaluated as part of the study. Data on growth hormone (GH) were included only postoperatively, since very few clinicians investigated or documented a preoperative GH deficiency.

### 2.2 Histopathological and Immunohistochemical Evaluation

In accordance with routine clinical methods, tissue samples from surgery were histopathologically evaluated, and the tumours were classified by an experienced neuropathologist according to the current WHO classification, which is based on the immunohistochemical expression of anterior pituitary hormones and the transcription factors SF1, PIT1 and TPIT. IHC for SF1 and PIT1 has been routinely performed on all specimens in the Department of Pathology since 2014, and IHC for all three TFs has been performed since 2017. Two of three tumours that met the criteria for so‐called null cell tumours were reevaluated, and the IHC work‐up was completed with an antibody against TPIT to allow cell lineage‐based classification; however, this did not change the classification of null cell tumours. Information on the mitotic count and p53 and Ki67 proliferation index levels was obtained from pathology reports.

Immunohistochemical analyses with antibodies against anterior pituitary hormones, pituitary‐specific TFs, and the level of the proliferation markers Ki67, the mitotic count, and the level of p53 were performed according to standard clinical diagnostic protocols [27].

### 2.3 Radiological Evaluation

The latest preoperative MRI examination for each patient was reevaluated by an experienced neuroradiologist (JW) to assess tumour volume and growth via the modified Knosp classification, where grade ≥ 3 was considered indicative of invasive growth [28]. The equation ((width × height × depth)/2) was used when calculating tumour volume [29].

### 2.4 Definitions of Anterior Pituitary Deficiency

Deficiencies in each of the pituitary axes were defined on the basis of a combination of the clinical evaluation performed at the pre‐ and postoperative visits, ongoing or initiated replacement therapy, and/or a retrospective evaluation of sex‐ and age‐matched laboratory results from pre‐ and postoperative visits. When appropriate, stimulation tests of the GH and hypothalamic–pituitary–adrenal (HPA) axes were performed. Different cut‐offs and reference ranges were used depending on which analysis method was used during the time for testing. The retrospective evaluations of laboratory results were based on current national and international guidelines. Data about preoperative GH levels were not included since stimulation tests were usually performed only postoperatively.

Guidelines at that time defined a deficiency on the HPA axis as a serum cortisol below 450 nmol/L or 550 nmol/L after a Synachten stimulation test, depending on which laboratory analysis method was used during the period for testing. Thyroid stimulating hormone (TSH) deficiency was defined as free thyroxin or free thyroxine and free triiodothyronine below reference range without a compensatory increase in TSH. Follicle stimulating hormone/luteinizing hormone (FSH/LH) deficiency in men was defined as low testosterone without a compensatory increase in FSH/LH. Premenopausal women with amenorrhea and low estradiol and FSH/LH when not on oral contraceptives and postmenopausal women with FSH/LH below the reference range for the postmenopausal phase were defined as deficient. Postoperatively, a low insulin‐like growth factor 1 (IGF‐1) value in combination with three other deficient pituitary axes, or deficiency according to a standard GH stimulation test classified a patient as GH‐deficient.

### 2.5 Statistical Analyses

Fisher's exact test or the Mann–Whitney U test was used to investigate any significant differences between SCTs and SGTs. Binary logistic regression was used to examine possible predictors of the dichotomous outcome of pituitary failure among patients with SGTs and those with SCTs, and *p* < 0.05 was considered to indicate statistical significance. IBM SPSS Statistics version 27 was used for all the statistical analyses.

## 3 Results

### 3.1 The Whole Study Population

Among the entire study population, 72% of patients (73/101) were classified as having SGTs, followed by 18% of patients (18/101) classified as having SCTs (Table 1).

**TABLE 1:** NF‐PitNET subtypes in the study population.

Subtype	Number of patients *N* = 101
SF1 cell lineage	73
TPIT cell lineage^a^	18
PIT1 cell lineage	2
PIT1 and SF1 cell lineage	2
Null cell tumour	3
Necrotic tissue (not assessable)	3

*Note:* SF1 steroidogenic factor 1 (SF1) is expressed in cells from the gonadotroph cell lineage, TPIT (The T‐Box family member TBX19 is a transcription factor for the corticotroph cell lineage), PIT1 (pituitary‐specific positive transcription factor 1 is a TF for the somatotroph, lactotroph and thyrotroph cell lineages).

^a^ The subtype SCT includes one Crooke's cell adenoma.

In terms of Ki67 staining, ≥ 3% Ki67 staining was observed in 9% (9/98) of the patients, ≥ 5% Ki67 staining was observed in 4% (4/98), and no patient exhibited ≥ 10% Ki67 staining. Ten percent (10/96) of samples were p53 positive, with more than 10 distinctly positive cells per 10 high‐power fields, and 5% (5/99) had a mitotic count above 2/10 high‐power fields [30].

The study population consisted of 65% (66/101) men and 35% (35/101) women (Table 2). The number of patients with inaccessible clinical data is shown in (Table 2). The median age at the time of surgery was 63 (IQR: 52–71) years. Among all the patients (*N* = 101), the preoperative median tumour volume was 6.5 (IQR: 3.3–10.5) cm^3^, and a Knosp grade ≥ 3 was observed in 29% (29/101) of the patients. The median postoperative follow‐up time was 2.2 years (range 0.1–5.7 years). Preoperatively, 23% (20/88) were deficient in the HPA axis, 48% (39/82) were deficient in the TSH axis, and 57% (43/76) were deficient in the LH/FSH axis. In total, 59% (57/96) of the patients were deficient in at least one pituitary axis. There were no significant differences between men and women regarding age at the time of surgery, preoperative tumour volume, Knosp grade ≥ 3 preoperative prolactin elevation, or any pituitary failure.

**TABLE 2:** Preoperative characteristics and postoperative pituitary failure in the study population.

	All subtypes	SGT	SCT	*p* (SCT vs. SGT)
Sex				0.28
Men, % (*n*)	65 (66)	66 (48)	50 (9)	
Women, % (*n*)	35 (35)	34 (25)	50 (9)	
Age at surgery, years, median (IQR)	63 (52–71)	65 (55–73)	48 (36–68)	0.003
Tumour volume, cm^3^, median (IQR)	6.5 (3.3–10.5)	6.6 (3.3–10.6)	5.0 (3.4–8.3)	0.54
Preop. pituitary failure^a^, % (*n*)	59 (57)	61 (42)	39 (7)	0.11
Missing data preop. pituitary failure, *n*	5	4	0	
Postop. pituitary failure^a^, % (*n*)	65 (61)	63 (45)	73 (11)	0.56
Missing data postop. pituitary failure, *n*	7	2	3	
Prolactin elevation, % (*n*)	56 (50)	54 (34)	56 (10)	1.0
Missing data prolactin elevation, *n*	11	10	0	
Knosp ≥ 3% (*n*)	29 (29)	26 (19)	39 (7)	0.38

*Note:* Knosp grading according to growth and volume appearance on MRI.

Abbreviations: SCT, silent corticotroph tumour; SGT, silent gonadotroph tumour.

^a^ Pituitary failure in ≥ 1 hormone axis.

### 3.2 Silent Corticotroph Tumours and Silent Gonadotroph Tumours

**FIGURE 1:**
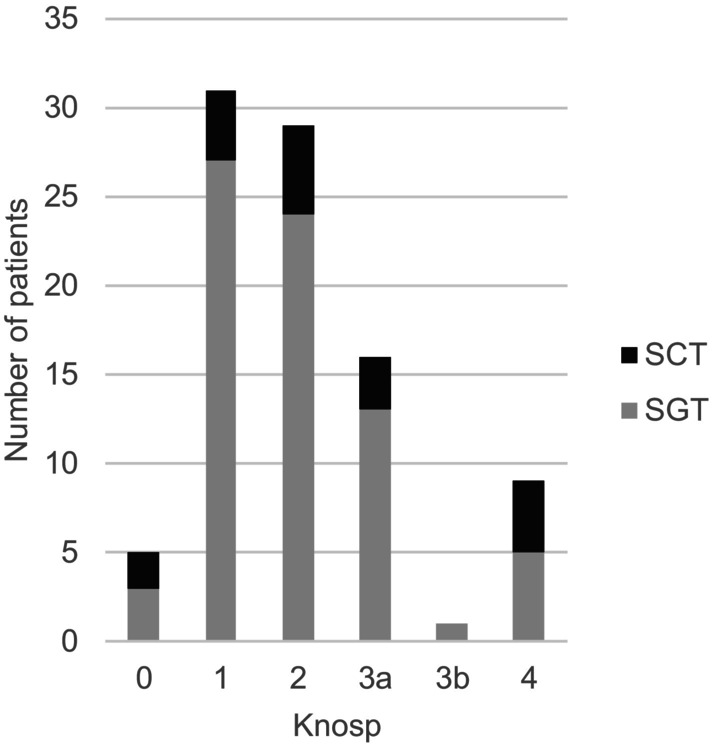
Knosp grading according to cell lineage for patients with SCTs (silent corticotroph tumours) and SGTs (silent gonadotroph tumours).

When the subgroups diagnosed with SGTs and SCTs were investigated, the population diagnosed with SGTs consisted of 66% (48/73) men, and the SCT population consisted of 50% (9/18) men, meaning that the male/female ratio among patients with SCTs was 1:1, whereas the ratio among patients with SGTs was 2:1 (*p =* 0.28). Patients with SCTs were younger, with a median age at the time of surgery of 48 years (IQR: 36–68) years, than patients harbouring SGTs, with a median age at the time of surgery of 65 years (IQR: 55–73) years (*p* = 0.003). No statistically significant differences were observed in the male/female ratio, preoperative tumour volume, proportion with Knosp grade ≥ 3 (Figure 1), or elevated prolactin level between the groups (Table 2).

In total, six patients (five SGT patients and one SCT patient) received radiotherapy postoperatively, and four patients (three SGT patients and one SCT patient) underwent reoperation.

### 3.3 Pituitary Failure in SCT and SGT Patients

Patients who had preoperative pituitary failure were significantly older at the time of surgery (*p* = 0.01) and more often had elevated preoperative prolactin levels (*p* = 0.01) than patients without preoperative pituitary failure. In patients with preoperative pituitary failure, the median tumour volume was 7.1 (IQR: 4.1–11.5) cm^3^, whereas in patients without preoperative pituitary failure, it was 4.5 (IQR: 3.1–8.5) cm^3^, *p =* 0.05. The difference between patients who had SGT or SCT in the presence of preoperative pituitary failure was not statistically significant (Table 2).

**FIGURE 2:**
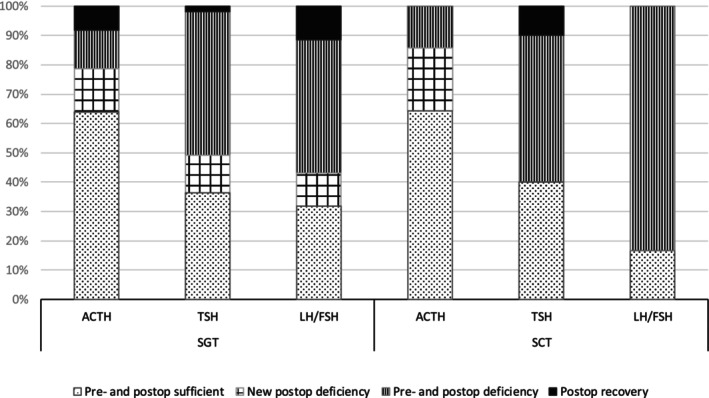
The proportion of SGT (silent gonadotroph tumour) and SCT (silent corticotroph tumour) patients with pre‐ and postoperative deficiency on each pituitary axis.

Postoperative pituitary failure, including GH deficiency, was observed in 65% (56/86) of patients; five patients lacked postoperative data on pituitary function. Patients with postoperative pituitary failure had significantly greater preoperative tumour volumes (*p* = 0.002). No differences in age at the time of surgery (*p =* 0.08), Knosp grade ≥ 3 (*p* = 0.8), sex (*p =* 0.49) or elevated preoperative prolactin levels (*p =* 0.81) were detected compared with patients with intact pituitary function. There was no significant difference in the incidence of postoperative pituitary failure between patients with SGTs and SCTs (*p =* 0.56) (Figure 2).

The binary logistic regression, which included the variables of age at surgery, sex, preoperative tumour volume, Knosp grade ≥ 3, preoperative prolactin levels and SCT (*n* = 77, SGT 62 and SCT 15), revealed that harbouring a SCT, compared with harbouring a SGT, was associated with a significantly increased likelihood of having postoperative pituitary failure (OR 6.4 (CI: 1.20–34.42), *p* = 0.03). Older age at the time of surgery and greater preoperative tumour volume were associated with an increased likelihood of having postoperative pituitary failure (OR 1.07 (CI: 1.02–1.12), *p* = 0.01 and OR 1.17 (CI: 1.04–1.32) *p* = 0.01, respectively) (Table 3). Collinearity diagnostics did not indicate problematic multicollinearity among the predictors (data not shown). Having a SCT did not increase the probability of preoperative pituitary failure (data not shown). There were no statistically significant differences regarding gender, age at surgery, preoperative tumour volume and prolactin elevation, KNOSP ≥ 3 and harbouring a SCT between the group included in the binary logistic regression and those who were not (*n* = 14) due to missing data on the variables used in the regression analyses.

**TABLE 3:** Effects of harbouring a SCT, gender, age at surgery, preoperative tumour volume, Knosp ≥ 3 and preoperative prolactin elevation on postoperative pituitary failure.

	B	S.E.	Wald	Sig.	Exp (B)	95% CI	
						Lower	Upper
SCT vs. SGT	1.86	0.86	4.70	0.03	6.41	1.20	34.42
Sex	−0.96	0.62	2.45	0.12	0.38	0.11	1.28
Age at surgery	0.07	0.02	7.73	0.01	1.07	1.02	1.12
Preop. tumour volume	0.16	0.06	6.50	0.01	1.17	1.04	1.32
Knosp ≥ 3	−0.19	0.67	0.08	0.78	0.83	0.22	3.07
Preop. prolactin elevation	0.47	0.57	0.69	0.41	1.60	0.53	4, 86

*Note:* Knosp grading of growth behaviour according to appearance on magnetic resonance imaging.

Abbreviations: SCT, silent corticotroph tumour; SGT, silent gonadotroph tumour.

## 4 Discussion

In logistic regression analysis, investigating potential predictors, harbouring a SCT, being older at the time of surgery, and having a larger preoperative tumour volume were factors associated with an increased likelihood of postoperative pituitary failure among patients with SCT and SGT.

In this study, SCT patients were significantly younger at the time of surgery, which is notable, since the SCT group neither had larger tumours nor more frequent preoperative hormone deficiencies than the SGT group. In some earlier studies, SCT patients were described as younger than other NF‐PitNET patients [22]. Unfortunately, from our data, or in general, it is not possible to conclude whether the SCTs grew more rapidly, prompting surgery, than the SGTs, as a speculative explanation. It has also been described that SCT patients more often have headaches at the time of diagnosis [25], amenorrhea and cranial nerve palsy [31]. Whether these symptoms, at least the latter, might prompt patients to seek healthcare and undergo MRI earlier is unclear. From our data we were not able to conclude which symptoms SCT patients had at diagnosis nor to further investigate biological, genetic, and environmental mechanisms, which are needed to understand the described age difference.

The proportion of patients with Knosp grade ≥ 3 among the SCT patients was 39% versus 26% among the SGT patients, which was not statistically significant in this cohort, but a different growth behaviour cannot be ruled out.

Harbouring a SCT increases the likelihood of postoperative pituitary failure, although this finding should be interpreted cautiously since the SCT group is small and the statistical confidence interval is wide (CI 1.20–34.42). However, other studies are in line with our results [22, 24, 32]. The median follow‐up time was 2.2 years; late onset pituitary failure as well as recovery from pituitary failure has been described earlier but the majority of the changes regarding hormonal status occur within the first postoperative years [16]. The increased likelihood of postoperative pituitary failure in SCT patients implies that these patients ought to be informed about symptoms associated with pituitary failure and they might also benefit from more frequent hormonal evaluations.

Increasing tumour volume and age were also predictors of postoperative hypopituitarism, and although not the focus of this study, somewhat surprisingly, they are not established risk factors for this postoperative result. Some, but not all, studies support such correlations [16, 33].

In a recent study, a weak correlation between serum prolactin levels and maximum tumour diameters in patients with NF‐PitNETs was described [34]. In our study, there was no significant difference in preoperative tumour volume between patients with and without elevated preoperative prolactin levels, and there was no histological evidence of lactotroph tumours. We interpreted the elevated prolactin levels as a stalk effect despite the similar tumour volumes. Why preoperative hyperprolactinemia is a risk factor for preoperative hypopituitarism is unclear. In a recent study, high intraoperative intrasellar pressure was significantly associated with preoperative hyperprolactinemia and TSH deficiency, but no significant difference in the ACTH axis was detected [35].

Previous studies revealed a greater proportion of women than men harbouring SCTs [18]; in one study, 94.3% (99/105) of all SCTs were observed in women [36], which is consistent with the prevalence of Cushing's disease [37]. This female overrepresentation was not confirmed in our study, nor did we observe a significant sex difference when we compared the proportion of women with SCTs (50%) with that of women with SGTs (34%), which might have been observed in a larger study population.

SCTs are described as potentially more aggressive in terms of invasive growth and recurrence [11], but studies are heterogeneous. In a systemic review and meta‐analysis, the recurrence rate ratio in SCTs was not higher compared with that of other NF‐PitNETs, but the classification was not based on TFs [38]. The present study, with a median postoperative follow‐up of 2.2 years, was designed primarily for clinical behaviour before and after surgery; however, within the study time frame, six patients (five SGT patients and one SCT patient) received radiotherapy postoperatively, and four patients (three SGT patients and one SCT patient) underwent reoperation.

The strength of this study is that all NF‐PitNETs were classified on the basis of the immunohistochemical analysis of anterior pituitary hormone and pituitary‐specific transcription factor expression, as recommended by the current WHO classification. All the surgeries were primary surgeries performed by one experienced neurosurgeon. If a patient was subjected to a second intervention that could affect pituitary function, such as radiotherapy or reoperation, data from after the intervention were excluded from the analysis. One limitation is the size of the study population; even though the proportion of patients with SCTs (18%) in this study is consistent with earlier studies [10], there is a class imbalance that calls for continuous use of transcription factors when classifying adenohypophysial tumours to enable future studies with larger cohorts.

## 5 Conclusion

In this study, we demonstrated that compared with SGTs, SCTs increase the likelihood of postoperative pituitary failure, which was not explained by preoperative pituitary failure or tumour volume; i.e., we did not find support for more aggressive clinical behaviour preoperatively in this subgroup. However, why patients with SCTs are younger at the time of surgery than patients with SGTs remains to be further investigated. The correlation between NF‐PitNET subtypes and clinical presentation and outcome is an additional argument for the use of both anterior pituitary hormones and transcription factors to classify adenohypophysial tumours.

## Author Contributions

**Nasrin Al‐Shamkhi:** conceptualisation, planning, data collection, data analysis, writing original draft, editing, approval of final draft. **Britt Edén Engström:** planning, methodology, data collection, review and editing, approval of final draft. **Olafur Gudjonsson:** planning, review, approval of final draft. **Johan Wikström:** methodology, data collection, review and editing, approval of final draft. **Olivera Casar‐Borota:** planning, methodology, data collection, review and editing, approval of final draft. **Eva Rask:** conceptualisation, planning, methodology, data collection, review and editing, approval of final draft.

## Funding

Nasrin Al‐Shamkhi and Eva Rask have received grants from the Swedish state under the agreement between the Swedish government and the county councils (ALF‐agreement) from Region Örebro County. Olivera Casar‐Borota was supported by the Swedish Cancer Society (grant number 190157 Fk) and by the grant from the Swedish state under the agreement between the Swedish government and the county councils (ALF agreement).

## Ethics Statement

All included patients gave informed, written consent to participate in research as part of the Uppsala Umeå Comprehensive Cancer Consortium (U‐CAN) project. Most of the patients were asked for consent when admitted to the hospital for surgery or, in some cases, through an information letter with a request for written consent sent to the patient postoperatively. The study was approved by the Ethics Committee in 2018 (Dnr 2018/053) and 2020 (Dnr 2019/04429). The study was preregistered in the database FoU i Sverige (researchweb.org) project number 274332.

## Conflicts of Interest

The authors declare no conflicts of interest.

## Data Availability

Datasets generated and/or analysed during this study are available from the corresponding author on reasonable request. The data are not publicly available due to privacy or ethical restrictions.
